# Consistent apparent Young’s modulus of human embryonic stem cells and derived cell types stabilized by substrate stiffness regulation promotes lineage specificity maintenance

**DOI:** 10.1186/s13619-020-00054-4

**Published:** 2020-09-03

**Authors:** Anqi Guo, Bingjie Wang, Cheng Lyu, Wenjing Li, Yaozu Wu, Lu Zhu, Ran Bi, Chenyu Huang, Jiao Jiao Li, Yanan Du

**Affiliations:** 1 Department of Biomedical Engineering, Tsinghua-Peking Center for Life Sciences, MOE Key Laboratory of Bioorganic Phosphorus Chemistry and Chemical Biology, School of Medicine, Tsinghua University, Beijing, 100084 China; 2 School of Life Sciences, Tsinghua University, Beijing, 100084 China; 3 Department of Biomedical Engineering, McKelvey School of Engineering, Washington University in St. Louis, St. Louis, 63130 USA; 4 Institute of Systems Engineering, Academy of Military Sciences, Beijing, 100071 China; 5 Department of Dermatology, Beijing Tsinghua Changgung Hospital, School of Clinical Medicine, Tsinghua University, Beijing, 102218 China; 6 Kolling Institute, University of Sydney, Sydney, NSW 2006 Australia

**Keywords:** Apparent Young’s modulus, Human embryonic stem cells, Substrate stiffness, YAP, Cell fate

## Abstract

**Background:**

Apparent Young’s modulus (AYM), which reflects the fundamental mechanical property of live cells measured by atomic force microscopy and is determined by substrate stiffness regulated cytoskeletal organization, has been investigated as potential indicators of cell fate in specific cell types. However, applying biophysical cues, such as modulating the substrate stiffness, to regulate AYM and thereby reflect and/or control stem cell lineage specificity for downstream applications, remains a primary challenge during in vitro stem cell expansion. Moreover, substrate stiffness could modulate cell heterogeneity in the single-cell stage and contribute to cell fate regulation, yet the indicative link between AYM and cell fate determination during in vitro dynamic cell expansion (from single-cell stage to multi-cell stage) has not been established.

**Results:**

Here, we show that the AYM of cells changed dynamically during passaging and proliferation on substrates with different stiffness. Moreover, the same change in substrate stiffness caused different patterns of AYM change in epithelial and mesenchymal cell types. Embryonic stem cells and their derived progenitor cells exhibited distinguishing AYM changes in response to different substrate stiffness that had significant effects on their maintenance of pluripotency and/or lineage-specific characteristics. On substrates that were too rigid or too soft, fluctuations in AYM occurred during cell passaging and proliferation that led to a loss in lineage specificity. On a substrate with ‘optimal’ stiffness (i.e., 3.5 kPa), the AYM was maintained at a constant level that was consistent with the parental cells during passaging and proliferation and led to preservation of lineage specificity. The effects of substrate stiffness on AYM and downstream cell fate were correlated with intracellular cytoskeletal organization and nuclear/cytoplasmic localization of YAP.

**Conclusions:**

In summary, this study suggests that optimal substrate stiffness regulated consistent AYM during passaging and proliferation reflects and contributes to hESCs and their derived progenitor cells lineage specificity maintenance, through the underlying mechanistic pathways of stiffness-induced cytoskeletal organization and the downstream YAP signaling. These findings highlighted the potential of AYM as an indicator to select suitable substrate stiffness for stem cell specificity maintenance during in vitro expansion for regenerative applications.

## Background

The mechanical properties of cells and associated forces in the cell cytoskeleton are critical elements in mechanochemical signaling pathways, which play a major role in defining fundamental cell functions (Galbraith & Sheetz, 1998; Vogel & Sheetz, 2006) including stem cell differentiation (Chaudhuri & Mooney, 2012). Recently, the elasticity of live cells, which is a fundamental property of cell mechanics and determines the cells’ ability to sustain their shape when exposed to mechanical stimuli (Moeendarbary & Harris, 2014), has been reported to comprise complicated mechanical behavior including viscoelasticity and poroelasticity (Moeendarbary et al., 2013; Hu et al., 2017; Efremov et al., 2017). The elastic properties of a live cell has been classically, universally and sensitively measured by AFM and analyzed by the Hertz model to extract the Young’s modulus, which gives a general reflection of the cell’s mechanical properties (Rotsch & Radmacher, 2000). In this work, we defined the apparent elastic modulus of live cells (Dokukin et al., 2013; Collinsworth et al., 2002) directly measured by AFM as the apparent Young’s modulus (AYM).

AYM of live cells is mainly determined by the organization of cytoskeletal elements (Fletcher & Mullins, 2010), and varies among single cells within a population of the same cell type, as well as among different cell types (Butt et al., 2005). There is evidence that the AYM can be potentially used as a label-free indicator of cellular alteration or abnormalities during stem cell development (Yu et al., 2010; Titushkin & Cho, 2007) or disease pathogenesis (Iyer et al., 2009; Lekka et al., 2012). For instance, the lower rigidity of cancer cells was recently suggested as a marker for cancer diagnosis (Paszek et al., 2005; Lekka & Laidler, 2009). Other studies have suggested that the AYM may be used as an indicator of cell fate in specific cell types such as ovarian cancer cells (Wenwei et al., 2012) and mesenchymal stem cells (MSCs) (Collinsworth et al., 2002).

To provide optimal cell sources for applications in cell therapy (Li et al., 2010; Li et al., 2014) and establishment of disease models (Yan et al., 2017), it is essential to ensure that the precursor cell types could maintain their desired cell fate during the in vitro expansion process, including characteristics such as self-renewal capacity and maintenance of lineage specificity. As an example, human embryonic stem cells (hESCs) can grow almost indefinitely in vitro and maintain the capacity to differentiate into cells from all three germ layers. hESCs can be induced to differentiate into endoderm cells, which are multipotent and give rise to cells of the gastrointestinal tract (gut, liver, and pancreas), the respiratory system (lung and trachea), and the thyroid (Zorn & Wells, 2009). Endoderm cells can be further induced to differentiate into bipotent hepatoblasts, which give rise to hepatocytes and cholangiocytes with potential applications in stem cell therapy for acute liver diseases (Li et al., 2010) and in vitro cell or tissue models (Yan et al., 2017; Takebe et al., 2013; Camp et al., 2017). At each level of differentiation from hESCs to endoderm cells to hepatoblasts, poorly defined conditions during in vitro cell expansion may lead to the instability of lineage specificity, or ‘lineage infidelity’ (Ge et al., 2017), which may alter or remove the defining characteristics of these cell types and limit their usefulness for downstream applications. Recent studies have found that in addition to biochemical factors, biomechanical factors in the microenvironment including spatial confinement and extracellular matrix (ECM) stiffness have profound effects on directing cell behavior and fate (Gvaramia et al., 2017). These biomechanical influences can directly impact cell mechanical characteristics including cell stiffness (AYM) and cell shape, leading to changes in the maintenance of pluripotency in stem cells and/or their differentiation into specific lineages (Mathieu & Loboa, 2012). However, a primary challenge surrounding the application of this knowledge to in vitro cell culture is the ability to apply biophysical cues, such as modulating the substrate stiffness, to regulate AYM and thereby reflect and/or control stem cell lineage specificity for downstream applications.

In our previous work, we investigated the effects of substrate stiffness on cellular heterogeneity during cell expansion from the initial single-cell stage to the resulting multi-cell stage (Wang et al., 2016). We found that the level of variance in hESC heterogeneity in the single-cell stage (prior to in vitro proliferation) directly influenced the percentage of cells that could maintain stemness within hESC colonies in the multi-cell stage (after in vitro proliferation). Furthermore, substrate stiffness could modulate cell heterogeneity in the single-cell stage and contribute to regulating cell fate. However, the indicative link between AYM and cell fate determination during in vitro dynamic cell expansion (from single-cell stage to multi-cell stage) has not been established.

It has been previously proved that cells could sense rigidity cues from the substrate and respond through a series of coordinated behaviors, including focal adhesion formation (Pelham & Wang, 1997; Yang-Kao et al., 2003; Wei et al., 2008; Friedland et al., 2009) and cytoskeletal organization (Tony et al., 2005; Gupta et al., 2015), immediately after passaging onto a substrate with a specific stiffness. YAP, as an important transcriptional factor downstream of the cytoskeletal signaling pathways, is speculated to play an vital role in cell mechanotransduction (Dupont et al., 2011). Cytoskeletal changes involving structural changes in contractile filamentous actin (F-actin) in response to substrate stiffness determine YAP nuclear localization and activity through unidentified molecular effectors (Das et al., 2016). Given the roles of YAP in regulating stem cell self-renewal, differentiation, apoptosis, and cell fate determination (Ian et al., 2010; Kyung-Kwon & Shin, 2012; Hsiao et al., 2016), it may be an essential element in AYM-related mechanotransduction.

In this study, we established a comprehensive sequential correlation among substrate stiffness, AYM, and cell fate. Specifically, we investigated the previously unanswered questions of: (1) How does AYM change dynamically in different cell types in response to the same change in substrate stiffness? (2) How does the dynamic AYM change pattern in specific cell types correlate with their downstream fate? (3) What are the potential mechanisms involved in cell fate determination when AYM is regulated through substrate stiffness? We hypothesized that during cell expansion, the AYM of the daughter cells needs to be regulated by substrate stiffness to replicate as closely as possible the AYM of the parental cells to ensure lineage specificity, and that these interactions are reliant on YAP-mediated pathways. To systematically investigate the relationships between AYM and substrate stiffness, we employed our previously developed PEGDA hydrogel system to fabricate a series of hydrogels with controlled stiffness, which could provide variations in mechanical stimulation only without accompanying changes in chemical stimulation (Wang et al., 2016). Our findings highlighted the significance of maintaining a consistent AYM in stem cells to preserve lineage specificity for cell-based applications, and provided a new perspective in regulating cell fate through AYM and the mechano-transductive pathways involved.

## Results

### AYM changes in epithelial and mesenchymal cell types after passaging onto substrates with different stiffness

**Fig. 1:**
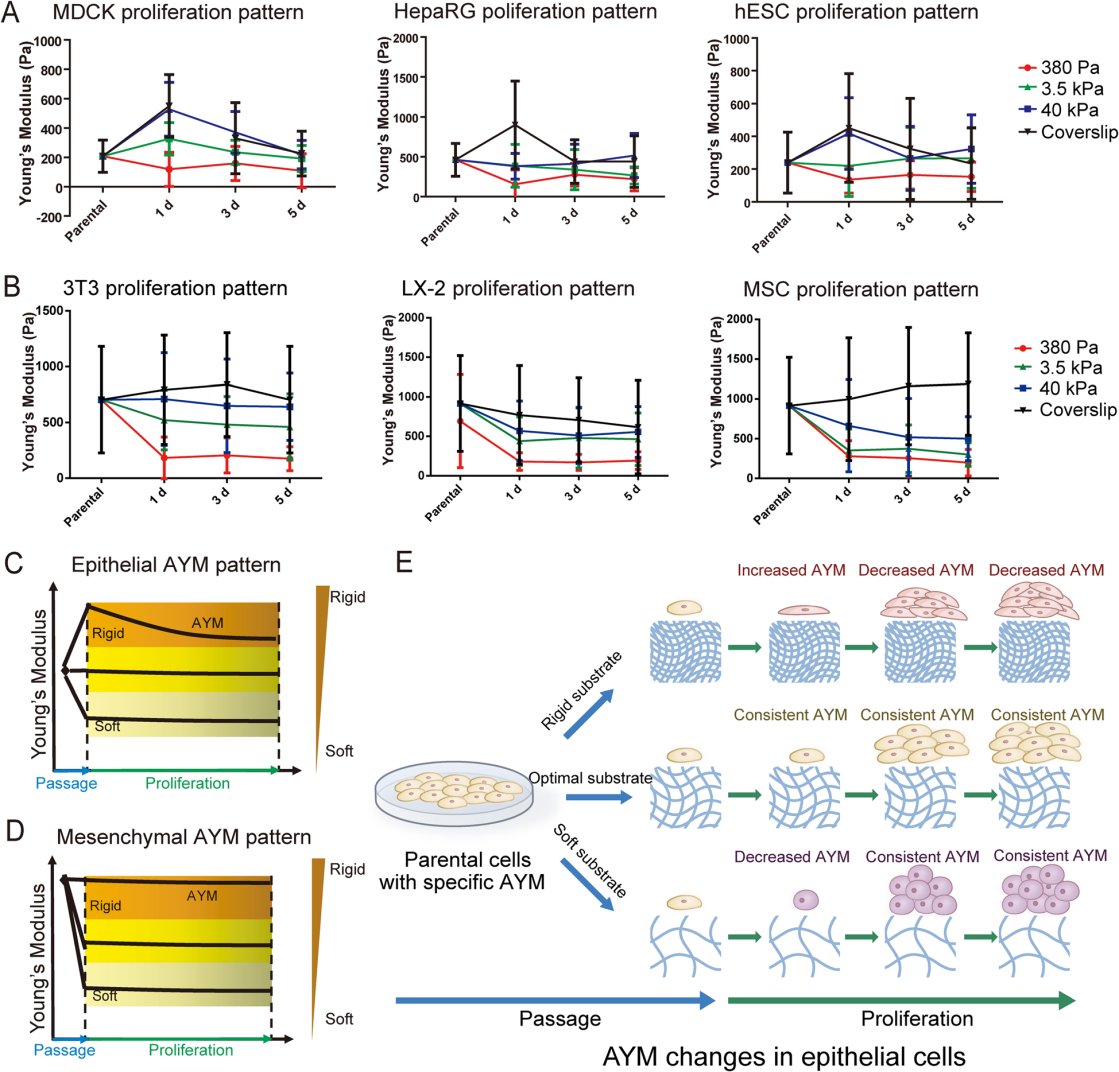
Dynamic AYM changes of epithelial and mesenchymal cell types on different substrate stiffness. **a** Epithelial (MDCK, HepaRG, hESCs) and **b** mesenchymal (LX-2, 3 T3, MSCs) cell types both shown dynamic changes in AYM following passaging and in vitro proliferation. **c**, **d** Dynamic changes in AYM in response to different substrate stiffness varies between epithelial and mesenchymal cell types. **e** Schematic of the pattern of AYM changes in epithelial cells during passaging and proliferation

We tested a series of epithelial (MDCK, HepRG and hESCs) and mesenchymal (3 T3, LX-2 and human adipose-derived MSCs) cell types to systematically investigate dynamic changes in AYM during in vitro culture on substrates with different stiffness (Fig. 1, S2, S3). The substrates used were PEGDA hydrogels with controlled stiffness of 380 Pa, 3.5 kPa and 40 kPa produced by regulating UV exposure time (Supplementary Fig. 1a), as well as glass coverslips. The micro-BCA assay showed that there were no significant variations in surface proteins among the selected groups of PEGDA hydrogels, indicating that there were no differences in protein conjugation among these substrates (Supplementary Fig. 1b).

All epithelial cell types showed distinct changes in AYM when passaged onto substrates with different stiffness (Fig. 1a, c). When cultured on rigid substrates, epithelial cells first showed a large increase in AYM at 1 day post-passage, followed by gradually decreasing AYM thereafter. On much softer substrates, epithelial cells first underwent a decrease in AYM at 1 day post-passage, and this decreased AYM was maintained thereafter. On a substrate with ‘optimal’ stiffness, which was 3.5 kPa for epithelial cells, the AYM of daughter cells was maintained consistently at the same level as the parental cells following passaging and in vitro proliferation. In contrast, the mesenchymal cell types showed different patterns of changes in AYM following passaging onto the same substrates (Fig. 1b, d). Compared to epithelial cells (Supplementary Fig. 2), all mesenchymal cell types had higher parental cell AYM (500–1000 Pa) (Supplementary Fig. 3), which was maintained after passaging onto rigid substrates. On softer substrates, the AYM decreased at 1 day post-passage and was maintained at the same level thereafter.

These results collectively indicated that dynamic changes in AYM occur in both epithelial and mesenchymal cell types following passaging onto substrates with different stiffness. However, AYM responses to changes in substrate stiffness were different between epithelial and mesenchymal cell types and were likely controlled through different mechanisms. Interestingly, while the AYM of epithelial cell types was partly influenced by the increased cell-cell contact at later stages of proliferation, as seen through their AYM changes on rigid substrates at 3–5 days, the AYM of mesenchymal cell types was not dramatically influenced by increased cell-cell contact during proliferation and seemed to only respond to changes in substrate stiffness. Considering these differences and the stiffness range of our hydrogel system, we chose to focus on epithelial cells and particularly hESCs and their derived cell types to investigate the sequential link between substrate stiffness, AYM and cell fate in the rest of the study.

### Cytoskeletal organization and focal adhesion formation in epithelial cell types on substrates with different stiffness

**Fig. 2:**
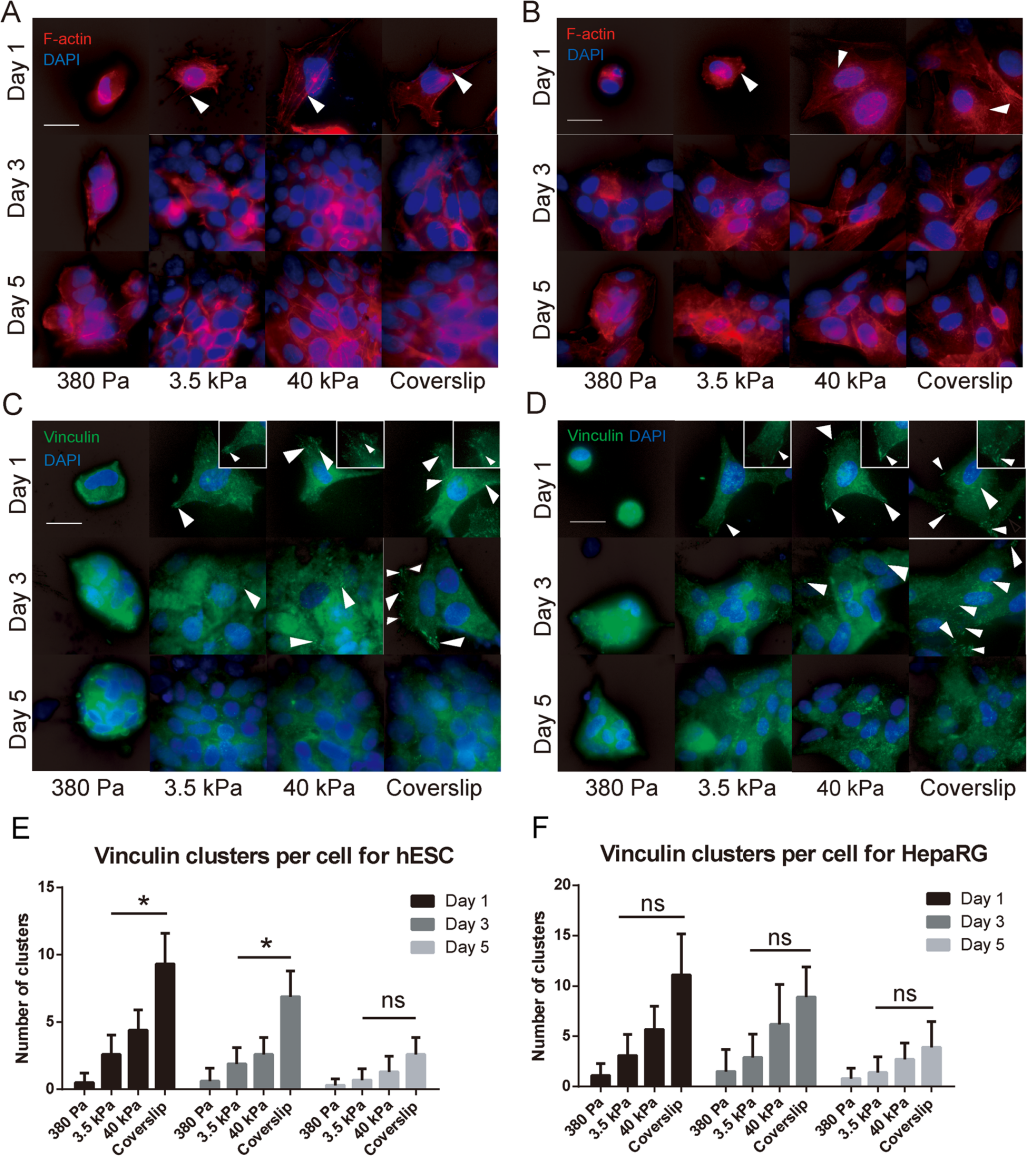
Cytoskeletal organization and focal adhesion formation in hESCs and HepaRG cells on different substrate stiffness. **a**, **b** Cytoskeletal organization (arrows = actin stress fibers) and **c**, **d** vinculin formation (arrows = elongated clusters, with locally enlarged vinculin clusters) in hESCs **a** and **c** and HepaRG cells **b** and **d** during culture on different substrates. Scale bars = 10 μm. **e**, **f** Quantitative analysis of vinculin clusters per cell in hESCs and HepaRG cells over the culture period. *P < 0.05

The cytoskeletal organization and formation of focal adhesions in response to substrate stiffness were analyzed in hESCs and HepaRG cells as representative human epithelial cell types. The cytoskeletal organization of these cells was evaluated through F-actin distribution. Cytoskeletal F-actin fibers of parental hESC colonies and HepaRG cells were rounded in shape, with cortical rings and few distinguishable stress fibers (Supplementary Fig. 4). After passaging, epithelial cells cultured on soft substrates (380 Pa) maintained a rounded shape throughout the culture period, with almost no distinguishable actin stress fibers (Fig. 2a, b). In contrast, cells cultured on rigid substrates (40 kPa hydrogels and coverslips) were well-spread with increasing formation of distinct actin stress fibers during proliferation. Cells cultured on the 3.5 kPa substrate showed some spreading with the formation of a few actin stress fibers during proliferation, and their cytoskeletal structure most closely replicated that of the parental cells.

Vinculin has a key role in focal adhesion formation and its expression was evaluated after the epithelial cells were passaged onto different substrates. The parental hESC colonies and HepaRG cells contained only a few vinculin clusters (Supplementary Fig. 4). Low vinculin expression was observed in both cell types after passaging onto the soft (380 Pa) substrate throughout the culture period (Fig. 2c, d). In contrast, many intense and elongated vinculin clusters formed on the rigid (40 kPa and coverslip) substrates immediately post-passage, but the number of clusters notably decreased over the 5-day culture period (Fig. 2e, f). Cells grown on the 3.5 kPa substrate showed dispersed vinculin distribution on the cell membrane, and the number of clusters was maintained at almost consistent levels throughout the culture period.

Collectively, these results suggested that while cytoskeletal organization and focal adhesion formation in epithelial cells were influenced by substrate stiffness, daughter epithelial cells growing on substrates with ‘optimal’ stiffness (i.e. 3.5 kPa) exhibit similar patterns of cytoskeletal organization and focal adhesion formation as the parental cells, and these patterns are consistently maintained during proliferation. What’s more, these patterns are consistent with the AYM change pattern, indicating AYM as an external manifestation of the link between substrate stiffness regulation and cytoskeletal changes in epithelial cells.

### Subcellular localization of YAP in epithelial cells on substrates with different stiffness

**Fig. 3:**
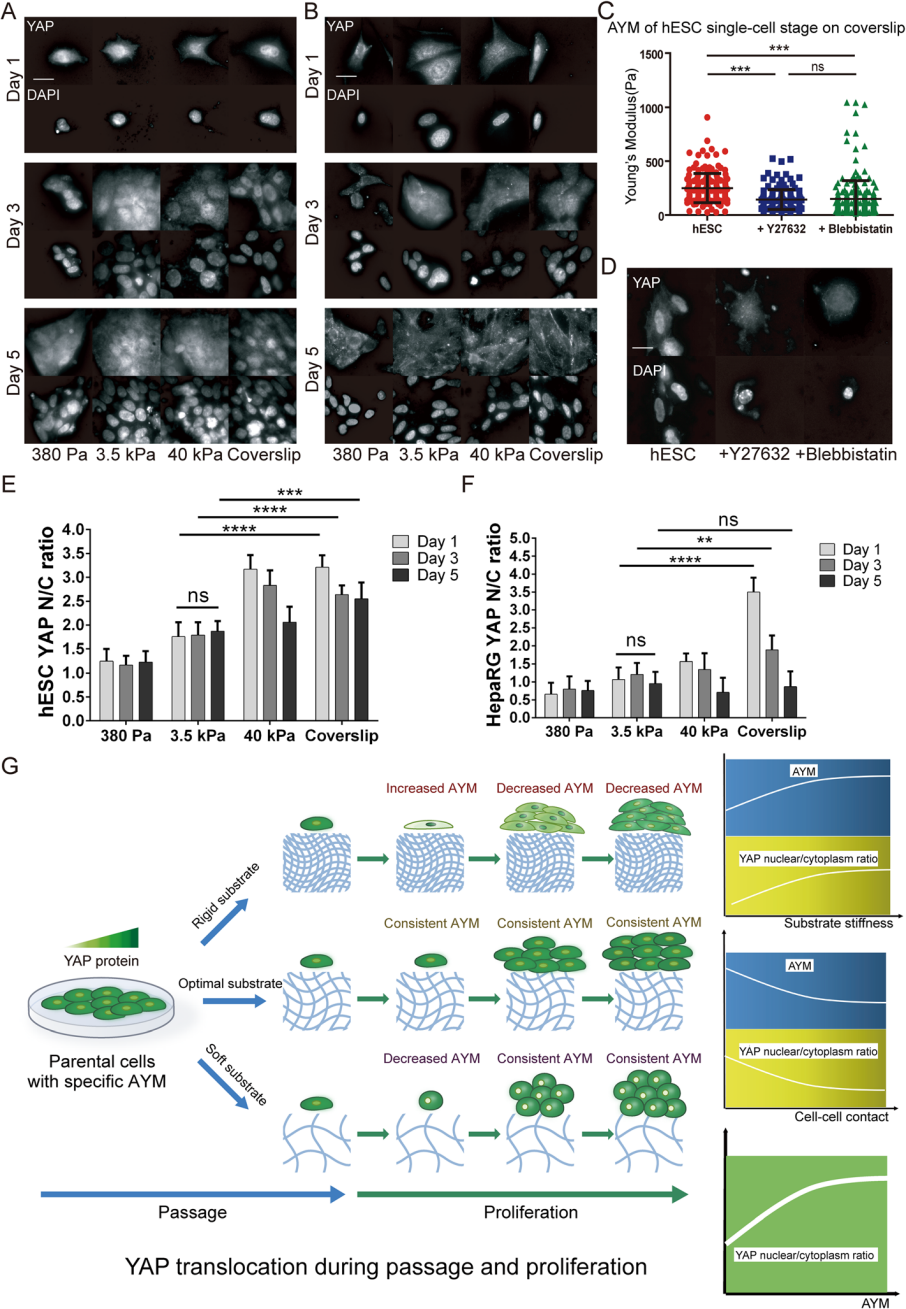
YAP subcellular localization in hESCs and HepaRG cells on different substrate stiffness. YAP translocation in **a** hESCs and **b** HepaRG cells during culture on different substrates. Scale bars = 10 μm. **c** AYM and **d** YAP translocation (nucleus to cytoplasm) of hESCs on coverslip after being treated with Y27632 and blebbistatin. Scale bar =10 μm. ***P < 0.001. Quantitative analysis of YAP nucleus/cytoplasm ratio in **e** hESCs and **f** HepaRG cells. **P < 0.01, ***P < 0.001, ****P < 0.0001. **g** Schematics of the relationships between AYM and YAP nucleus/cytoplasm ratio in response to substrate stiffness and cell-cell contact

YAP is a key mediator of cell mechanotransduction, where its translocation from the cytoplasm to the nucleus in response to increasing extracellular matrix rigidity activates the expression of target genes. The expression level of YAP was analyzed in hESCs and HepaRG cells during culture on substrates with different stiffness. When grown on a soft substrate (380 Pa), YAP in both cell types were mostly located within the cytoplasm over the culture period (Fig. 3a, b, e, f). When grown on rigid substrates (40 kPa, coverslips), YAP was initially localized in the nucleus, but its nucleus/cytoplasm ratio gradually decreased over the culture period. On the 3.5 kPa substrate, the nucleus/cytoplasm ratio of YAP was approximately maintained at a constant level during cell proliferation (Fig. 3a, b, e, f). The trends in nuclear translocation of YAP in response to changes in substrate stiffness were very similar to the changes observed in AYM.

To further investigate the relationship between AYM, cytoskeletal organization and YAP localization, parental hESCs (pre-passage) were grown on the most rigid substrate (coverslip) and treated with 50 μM Y27632 (ROCK signaling inhibitor) and 50 mM Blebbistatin (myosin II inhibitor) respectively for 24 h. These small molecule inhibitors disrupt cytoskeletal responses to high substrate stiffness (Supplementary Fig. 5). Correspondingly, treated hESCs maintained a low AYM despite being grown on a highly rigid substrate (Fig. 3c). Simultaneously, treated hESCs showed YAP translocation from the nucleus into the cytoplasm (Fig. 3d). These findings suggest that changes in AYM reflect dynamic changes in YAP subcellular localization, as a response to variations in substrate stiffness that lead to changes in cytoskeletal organization and focal adhesion formation. AYM can therefore be considered as being correlated with the YAP nucleus/cytoplasm ratio in the response of cells to substrate stiffness changes, as well as to the level of cell-cell contact (Supplementary Fig. 6) which increases during cell proliferation (Fig. 3g).

### Maintenance of pluripotency in hESCs on substrates with different stiffness

**Fig. 4:**
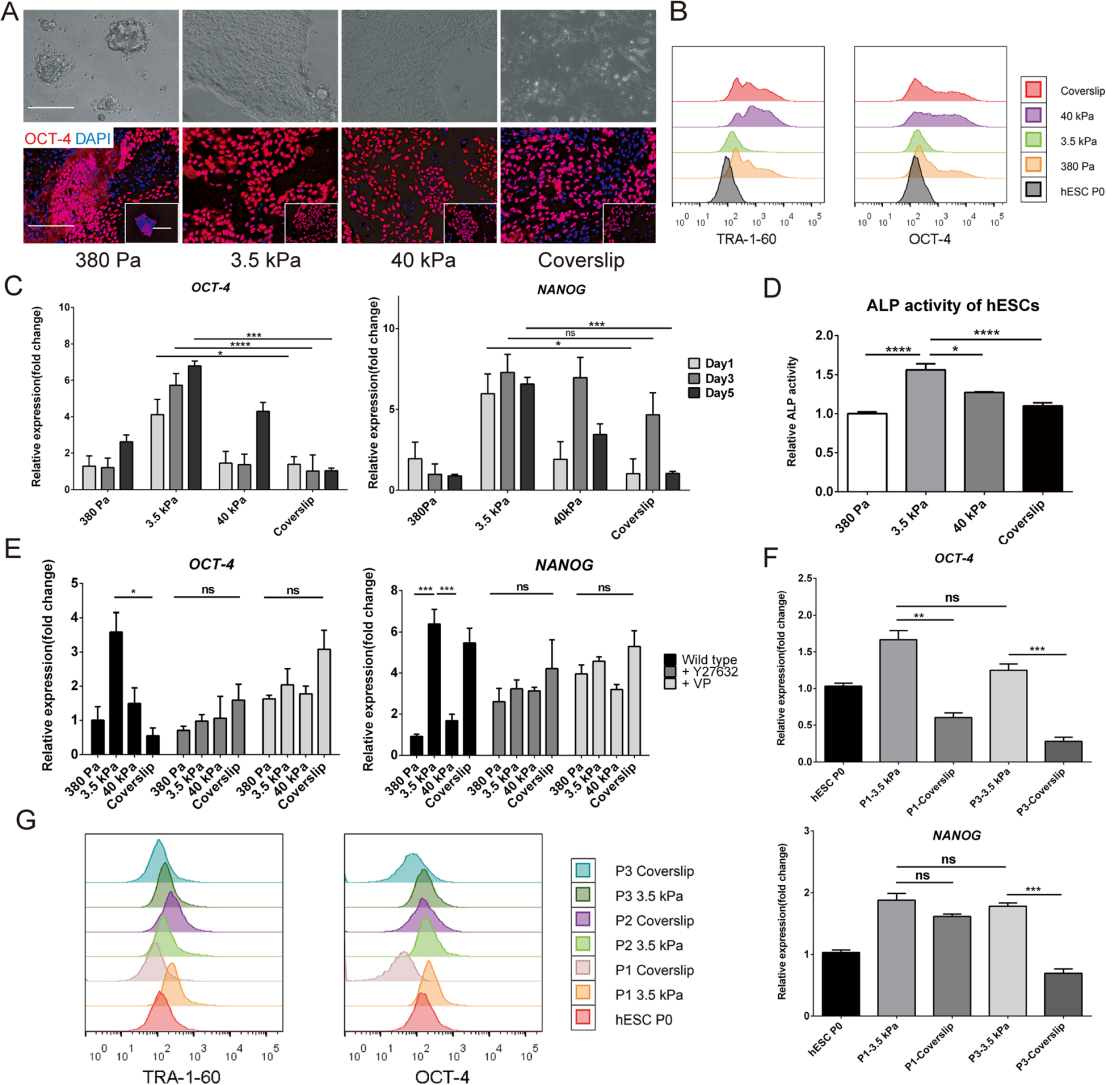
Maintenance of pluripotency in hESCs during in vitro culture on substrates with different stiffness. **a** Brightfield and immunostaining images of OCT-4 for hESCs cultured for 5 days on substrates with different stiffness. Scale bars = 200 μm. **b** FACS analysis of TRA-1-60 and OCT-4, **c** OCT-4 and NANOG expression, and **d** ALP relative activity in hESCs cultured for 5 days on substrates with different stiffness. **e** OCT-4 and NANOG expression in hESCs after being treated with Y27632 or Verteporfin. **f** OCT-4 and NANOG expression and **g** FACS analysis of TRA-1-60 and OCT-4 in up to 3 passages of hESCs on the 3.5 kPa substrate and coverslip. *P < 0.05, **P < 0.01, ***P < 0.001, ****P < 0.0001

The effect of changing substrate stiffness, which is accompanied by changes in AYM, on the maintenance of pluripotency in hESCs was investigated by analyzing their expression of pluripotency markers. After passaging onto hydrogels with different stiffness or coverslips and culturing for 5 days, protein expression of OCT-4 was the most prominent as well as a larger proportion of OCT-4 positive hESCs grown on the 3.5 kPa substrate compared to other groups (Fig. 4a). Fluorescence-activated cell sorting (FACS) analysis at day 5 also showed that hESCs cultured on the 3.5 kPa substrate was the most homogeneous pluripotent cell population expressing higher level of TRA-1-60 and OCT-4 compared to other groups (Fig. 4b). Similarly, ALP activity and expression of OCT-4 and NANOG, which represent the stemness maintenance of hESCs, were expressed at the highest level in the 3.5 kPa group at all time points tested (Fig. 4c, d).

When inhibitors were added to block cytoskeletal organization with 10 μM Y27632 within the whole 5 days’ culture, and block YAP nuclear translocation with 1uM Verteporfin for 2 h (removal of Verteporfin in the following 5 days’ culture) such that AYM in the hESCs could not change in response to substrate stiffness, the expression level of pluripotency markers OCT-4 and NANOG was no longer significant between groups unlike in the wild-type hESCs (Fig. 4e). Furthermore, by culturing on an optimal substrate stiffness of 3.5 kPa in this study, three consecutive passages of hESCs could all retain better pluripotency characteristics compared with those passaged on coverslips (Fig. 4f, g). These findings suggested that maintaining a consistent AYM by culturing on an optimal substrate stiffness was essential for conserving pluripotency in hESCs during passaging and proliferation.

### Maintenance of lineage specificity in definitive endoderm (DE) cells on substrates with different stiffness

**Fig. 5:**
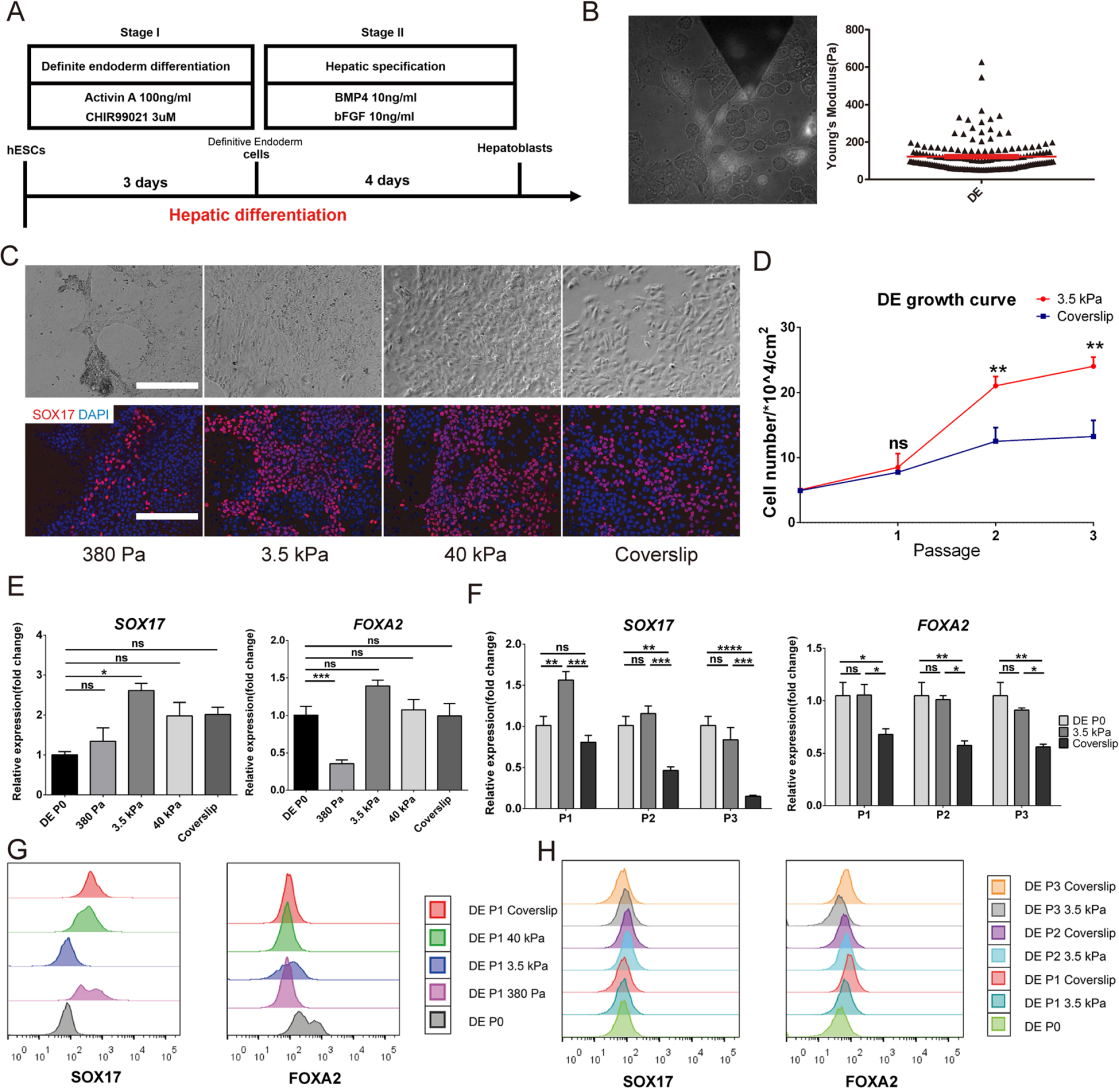
Maintenance of lineage specificity in DE on different substrate stiffness. **a** Schematic of the differentiation strategy for obtaining hESC-derived DE and hepatoblasts. **b** AYM measurement of parental DE with the SOX17-GFP reporter using AFM. **c** Brightfield and immunostaining images of SOX-17 for DE cultured for 5 days on substrates with different stiffness. Scale bars = 200 μm. **d** Growth curve of DE over 3 passages on the 3.5 kPa substrate and coverslip. SOX17 and FOXA2 expression **e** in DE cultured for 5 days on substrates with different stiffness and **f** in 3 passages of DE on the 3.5 kPa substrate and coverslip. FACS analysis of SOX17 and FOXA2 **g** in DE cultured for 5 days on substrates with different stiffness, and (**h**) in 3 passages of DE on the 3.5 kPa substrate and coverslip. *P < 0.05, **P < 0.01, ***P < 0.001, ****P < 0.0001

We proceeded to investigate whether the same link between substrate stiffness, AYM and maintenance of lineage specificity was present in stem cell types derived from hESCs. Specifically, DE were obtained during the process of hepatic differentiation of hESCs (Fig. 5a), which were used as the parental DE. The AYM of DE with the SOX17-GFP reporter was measured with AFM-total internal reflection fluorescence (TIRF), which provided a more accurate measurement of cell stiffness under its original microenvironment. The AYM of parental DE was measured to be ~ 127.6 Pa (Fig. 5b), which was within the range of epithelial cell stiffness (< 500 Pa) (Supplementary Fig. 2) and allowed the same substrate groups of 380 Pa, 3.5 kPa, 40 kPa hydrogels and coverslips to be applied for testing.

As a lineage-specific marker of DE, SOX-17 was the most highly expressed in cells cultured for 5 days on the 3.5 kPa substrate (Fig. 5c). Compared to parental DE and daughter cells passaged and cultured on other substrates, expression levels of the DE lineage specific markers SOX-17 and FOXA2 on the 3.5 kPa substrate remained the highest (Fig. 5e, g). In contrast, on very soft (380 Pa) or rigid (coverslip) substrates, a large proportion of cells have completely lost the expression of FOXA2.

When passaged three times, DE showed an elevated growth rate on the 3.5 kPa substrate compared to on coverslips (Fig. 5d), as well as better preservation of SOX17 and FOXA2 gene expression levels between passages (Fig. 5f). DE in the three passages showed an increase in fluorescence intensity through FACS analysis compared to parental cells when grown on 3.5 kPa substrates compared to on the coverslips (Fig. 5h). These findings suggest that substrate stiffness has a similar effect on controlling AYM and therefore lineage specificity maintenance of hESC-derived DE as it does on hESCs.

### Maintenance of lineage specificity in hepatoblasts (HBs) on substrates with different stiffness

**Fig. 6:**
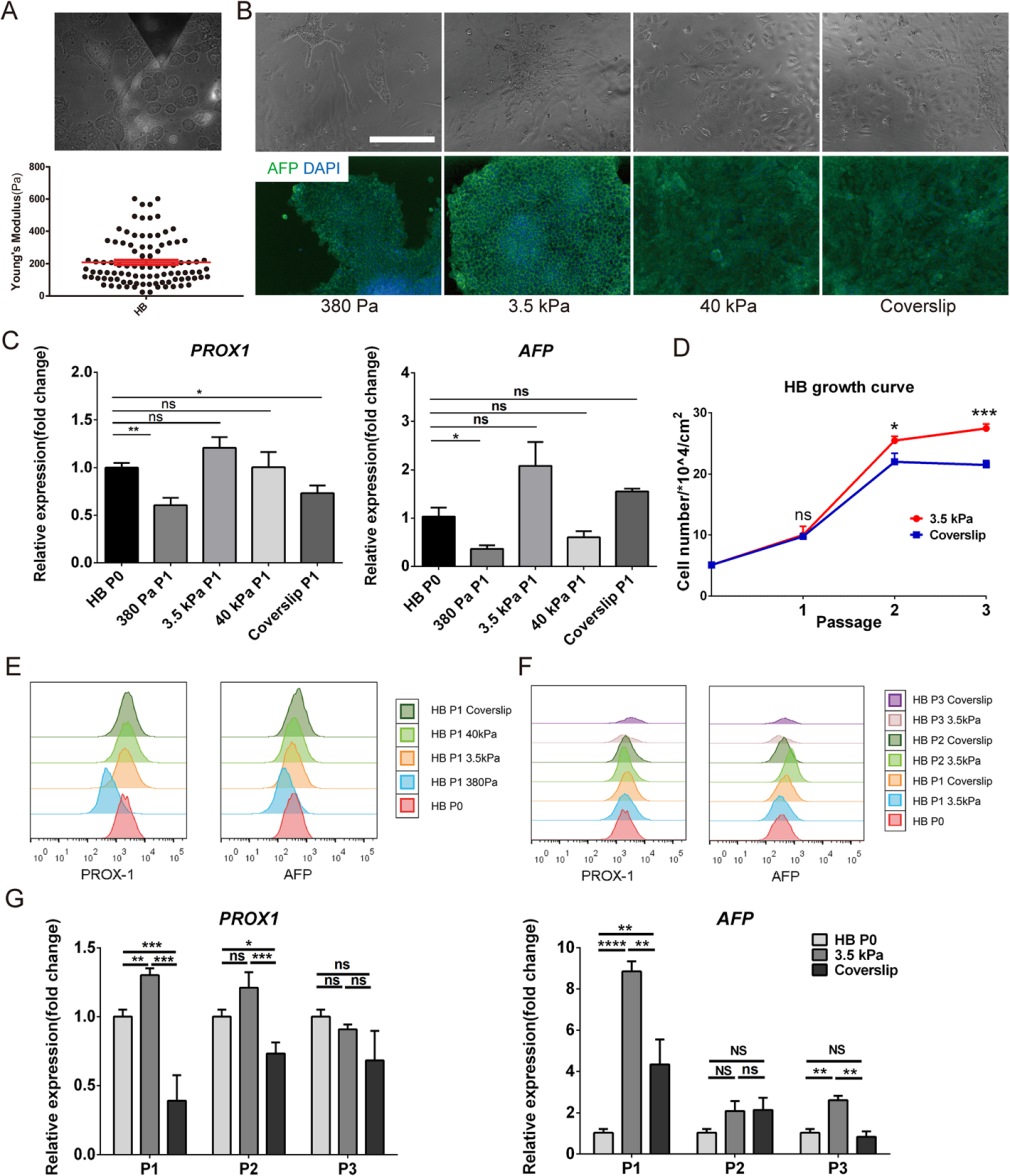
Maintenance of lineage specificity in HBs during in vitro culture on different substrate stiffness. **a** AYM measurement of parental HBs with the AFP-mCherry reporter using AFM. **b** Brightfield and immunostaining images of AFP for HBs cultured for 5 days on substrates with different stiffness. Scale bars = 100 μm. **c** PROX1 and AFP expression in HBs cultured for 5 days on substrates with different stiffness. **d** Growth curve of HBs over 3 passages on the 3.5 kPa substrate and coverslip. FACS analysis of PROX1 and AFP **e** in HBs cultured for 5 days on substrates with different stiffness, and **f** in 3 passages of HBs on the 3.5 kPa substrate and coverslip. **g** PROX1 and AFP expression in 3 passages of DE on the 3.5 kPa substrate and coverslip. *P < 0.05, **P < 0.01, ***P < 0.001, ****P < 0.0001

We further differentiated hESC-derived DE into HBs, which are downstream hepatic progenitors (Fig. 5a). The AYM of HBs with the AFP-mCherry reporter was measured to be 210 Pa with AFM-TIRF (Fig. 6a), which also fell into the range of epithelial cell types stiffness and allowed the same substrate groups to be applied as in previous experiments. HBs cultured for 5 days on the 3.5 kPa substrate achieved the highest expression of HB-specific markers, as shown through the percentage of AFP-positive cells (Fig. 6b) and expression of PROX1 and AFP (Fig. 6c) compared to other substrate groups. FACS analysis also showed better maintenance of the fluorescence intensity peak in the 3.5 kPa group relative to the parental cells compared to other groups (Fig. 6e).

HBs passaged three times on the 3.5 kPa substrate showed faster proliferation than those on coverslips (Fig. 6d), as well as better expression of PROX1 and AFP at each passage (Fig. 6f, g). Therefore, the lineage specificity maintenance of hESCs-derived HBs could be correlated with AYM stabilization by substrate stiffness regulation, similar to the effects observed in the hESCs and DE.

## Discussion

It is well established that cells sense and respond to substrate stiffness, which influences downstream cell activity and fate (Discher et al., 2005). However, the relation between substrate stiffness and AYM, a primary characteristic defining the mechanical properties of cells, and the influence of changing AYM on cell fate has not been systematically investigated. In this study, we demonstrate that (1) the same change in substrate stiffness causes different patterns of AYM changes in epithelial and mesenchymal cell types, (2) consistent AYM in epithelial cells in response to specific ‘optimal’ substrate stiffness reflects the maintenance of pluripotency in hESCs and lineage specificity in hESC-derived progenitor cell types, and (3) the effects of AYM changes on cell fate determination is at least partly regulated through mechanotransduction by the actin cytoskeleton and subcellular translocation of YAP. Importantly, our findings highlight that for epithelial cell types, better maintenance of lineage specificity can be achieved by controlling substrate stiffness to keep the AYM at a constant level during passaging and cell proliferation, which provides profound implications on optimizing culture conditions for cell therapy, disease modelling, and bio-mechanistic investigations.

Both cell-cell and cell-matrix interactions have critical roles in the mechanical regulation of cell fate. During the single-cell (pre-proliferation) stage where cell-cell interactions are absent, cell-matrix interactions dominate mechano-transductive cell fate regulation. At this stage, cells sense the substrate stiffness through integrin receptors on the cell membrane, and convert this into critical signals that regulate cell lineage specificity (Hansen et al., 1994). These cell-matrix interactions are weakened with cell proliferation, as increased cell density produces more cadherin mediated cell-cell interactions that begin to dominate cell mechanotransduction (Mertz et al., 2013). There is so far little evidence on the synergistic effects of these two types of interactions on regulating cell fate during in vitro expansion. In this study, we found that the AYM could indicate the coordination of cell-matrix and cell-cell interactions during passaging and proliferation, and served as an important ‘biomarker’ of mechanical regulation that correlated with cell fate determination. Notably, we observed a significant difference in dynamic AYM changes between epithelial and mesenchymal cell types in response to the same changes in substrate stiffness. In epithelial cells, AYM was regulated in the early stage of proliferation by cell-matrix interactions induced by substrate stiffness, and then by cell-cell interactions mediated by E-cadherin in the later stages. In mesenchymal cells, AYM regulation was dominated by cell-matrix interactions throughout cell proliferation, which matched the observations of others where fibroblasts could tune their internal stiffness to match their substrate within a defined range (Solon et al., 2007). Unlike epithelial cells, cell-cell interactions had no obvious effects on the fate of mesenchymal cells during passaging and proliferation (Raghu & Weinberg, 2015; Gheldof & Berx, 2013). Therefore, our findings illuminated the potential of AYM as label-free indicator to select appropriate substrate stiffness for stemness maintenance of epithelial-like stem cell types during long-term in vitro expansion. Compared to other biochemical indicators with the help of fluorescent antibodies or probes, parental stem cells AYM could be easily measured before passaged onto different substrate stiffness. Then the optimal substrate stiffness, on which daughter cells could maintain similar AYM as parental cells, would be selected for stem cell expansion in vitro. However, in mesenchymal cell types, cell fate determination likely occurs under more complicated mechanisms that only partly involve AYM and requires further investigation.

Substrate stiffness plays a powerful role in regulating cell behavior by modulating focal adhesion formation and cytoskeletal organization, leading to the conversion of mechanical cues into intracellular signals (Alenghat & Ingber, 2002; Ingber, 2006). YAP is a central player in the mechanosensing pathway and is a downstream transcriptional factor of cytoskeletal F-actin. Our results showed that the nucleus/cytoplasm ratio of YAP was directly influenced by focal adhesion formation and cytoskeletal organization in epithelial cells when grown on substrates with different stiffness. Changes in YAP nucleus/cytoplasm ratio were correlated with changes in AYM, and keeping these parameters at a constant level during proliferation by controlling the substrate stiffness had profound effects on maintaining pluripotency and/or lineage specificity in epithelial-lineage progenitor cells. When cultured on a rigid substrate such as a coverslip, the high substrate stiffness initially causes YAP translocation from the cytoplasm into the nucleus, which is externally manifested as an immediate increase in AYM. During cell proliferation on the rigid substrate, increased cell-cell contact leads to elevated E-cadherin expression that results in contact inhibition. This is mediated by the Hippo signaling pathway, which causes a cell density-dependent redistribution of YAP from the nucleus to the cytoplasm that inhibits cell proliferation and promotes apoptosis (Nam-Gyun et al., 2011), and manifests as a gradual decrease in AYM during proliferation. In addition, retention of YAP/TAZ complex in the cytoplasm favors the degradation of β-catenin and inhibits Wnt/β-catenin signaling, leading to downstream effects on cell fate including a loss of pluripotency in embryonic stem cells (Azzolin et al., 2014). YAP, as transcription coactivator, must bind to DNA -binding transcription factors such as TEAD to stimulate gene expression (Zhao et al., 2008), since YAP itself has no DNA binding activity. As reported before, YAP was inactivated with increased phosphorylation and cytoplasm translocation during embryonic stem cells differentiation. What’s more, YAP and TEAD knockdown lead to a loss of embryonic stem cells pluripotency, while ectopic expression of YAP prevented ES cells differentiation in vitro and maintains stem cell phenotypes even under differentiation conditions, suggesting that YAP and TEAD were required for ES cells pluripotency maintenance. Moreover, YAP bind to promoters of a large number of pluripotent marker genes (including Polycomb group (PcG) proteins, Nanog, Oct4 and Sox2) known to be important for stem cells pluripotency maintenance and stimulated their expression (Lian et al., 2010). In our study, hESCs could maintain AYM and YAP nucleus translocation at a constant level during passage and proliferation on the 3.5 kPa PEGDA-based hydrogel, indicating that the optimal substrate stiffness could maintain YAP activation for hESCs self-renewal, while fluctuations of YAP nucleus/cytoplasm ratio on other substrate stiffness indicating the loss of hESCs pluripotency.

Nevertheless, it should be noted that strategies for optimizing the maintenance of cell lineage specificity should not be limited to modulating the AYM. Extracellular matrix components and chemical stimuli in the microenvironment have critical roles in regulating cell fate, and their synergistic effect with mechanical factors should be explored in future studies.

## Conclusions

**Fig. 7:**
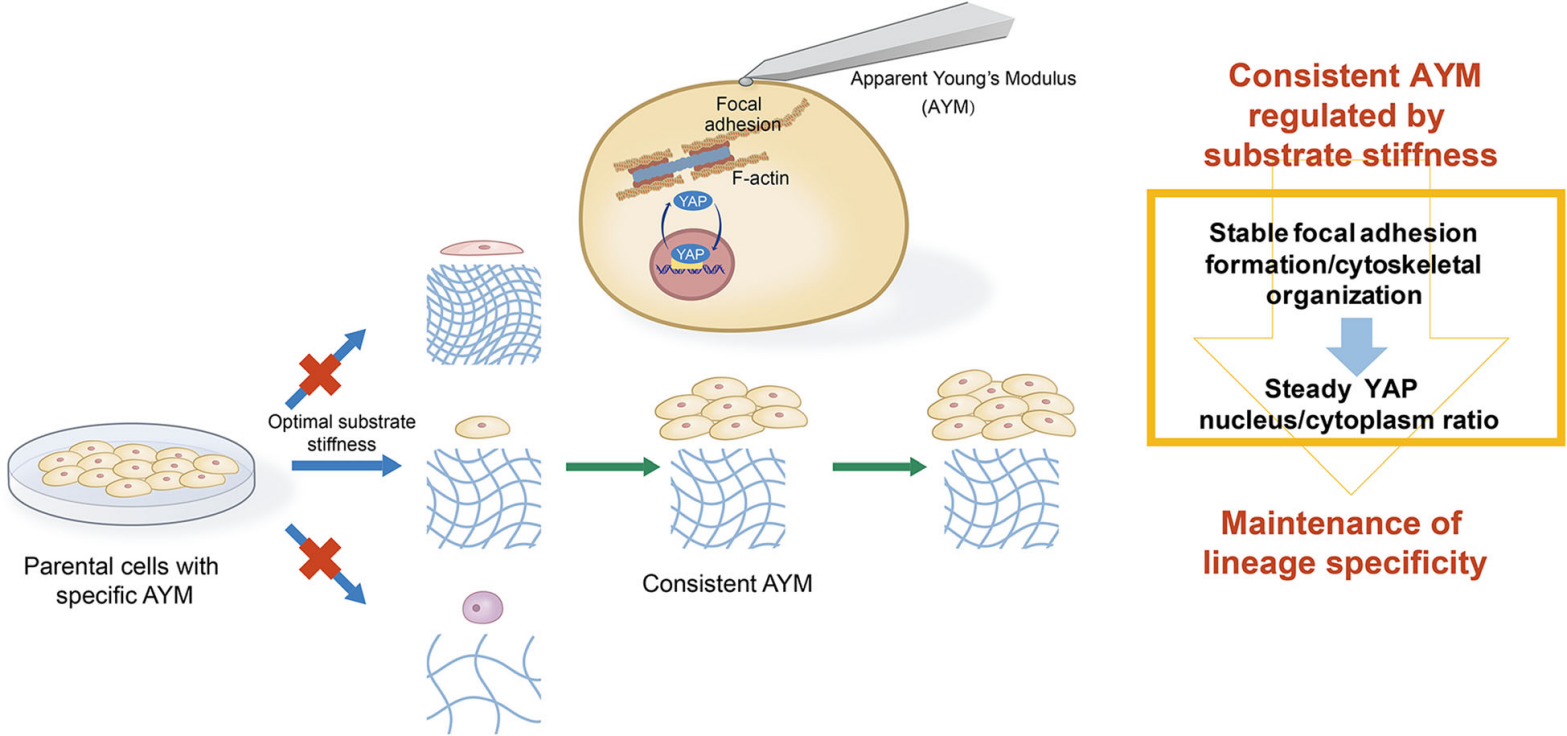
Schematic of the proposed link between substrate stiffness, AYM and cell fate

This study suggests that consistent AYM regulated by optimal substrate stiffness during passaging and proliferation could reflect and effectively contribute to hESCs and their derived progenitor cells lineage specificity maintenance (Fig. 7). Underlying mechanistic pathways involve stiffness-induced cytoskeletal organization and the downstream YAP nucleus/cytoplasm translocation. Our study provides novel insights for further investigation of mechanistic pathways in mechanobiology, and highlights the significance of AYM as indicators for suitable substrate stiffness selection to maintain stem cells specificity during in vitro large-scale expansion for regenerative applications.

## Methods

### Fabrication and functionalization of PEGDA-based hydrogel substrates

PEGDA hydrogels were prepared using methods modified from our previously published procedures (Wang et al., 2016). Briefly, a precursor solution was made by dissolving 10% w/v PEGDA, 0.5% w/v photoinitiator Irgacure D2959 (Insight High Technology Co. LTD, China) and 1% w/v N-acryloxysuccinimide (NAS) (J&K, China) in cold phosphate-buffered saline (PBS). The solution was photo-crosslinked by UV exposure (OmniCure SERIES 1500, Canada, 20 mW cm^− 2^) to form hydrogels between a 3-(trimethoxysilyl) propyl methacrylate (TMSPMA)-treated coverslip and an octadecyltrichlorosilane (OTS)-treated glass slide. Different hydrogel stiffness in this study was achieved by adjusting the UV irradiation time (380 Pa, 3.5 kPa and 40 kPa fabricated on TMSPMA-modified coverslips with diameters fitting commercial 24-well plates). Hydrogels were immersed in 75% ethanol for at least 1 h for sterilization and removal of un-crosslinked residuals. The hydrogels were functionalized for cell adhesion by coating the surface with different types of proteins at 4 °C overnight, that is, 0.1% gelatin (Sigma-Aldrich) dissolved in diH_2_O coated for HepaRG cells, 1:50 diluted vitronectin (Corning) for hESC-derived definitive endoderm (DE) cells, and 1:100 diluted Matrigel (hESC qualified, Corning) for hESCs and hESC-derived hepatoblasts. Surface coated proteins (0.1% gelatin coating) on hydrogels of different stiffness were characterized by micro-BCA assay kit (Beotime Biotechnology) according to manufacturer’s directions.

### Cell culture

Madin-Darby Canine Kidney (MDCK) cells, HepaRG cells (Life Technologies), and hESCs (H9 line), which represent epithelial cell types, and NIH/3 T3, human hepatic stellate cell line (LX-2) (Xiangya Hospital of Central South University, China), and human adipose-derived mesenchymal stem cells (MSCs), which represent mesenchymal cell types, were used in the subsequent experiments. All cell lines tested negative for mycoplasma. MDCK, 3 T3, LX-2, MSCs and HepaRG were cultured in a humidified 5% CO_2_ incubator (Thermo Fisher) at 37 °C. MDCK, 3 T3 and LX-2 cells were cultured in medium composed of high glucose Dulbecco’s modified Eagle medium (4.5 g/L glucose, Wisent, Canada) supplemented with 10% FBS (Wisent) and 1% penicillin-streptomycin (Wisent). MSCs were cultured in mesenchymal stem cell growth medium (BioWit Technologies) according to our previous study (Zeng et al., 2015). HepaRG cells were cultured in Williams’ E (Gibco) supplemented with 10% FBS, 1x Glutamax (Invitrogen), 10^− 7^ M dexamethasone (DEX) (Sigma-Aldrich), 5 μg/mL insulin (Aladdin) and 1% penicillin-streptomycin. H9 hESCs were cultured according to our previously published procedures (Yao et al., 2014). Briefly, cells were maintained under 5% CO_2_ at 37 °C on a feeder layer of X-ray inactivated mouse embryonic fibroblast (MEF) cells and seeded at a density of 2 × 10^5^/cm^2^. The medium used for hESCs was KO-DMEM (Gibco) supplemented with 20% (v/v) KSR (Invitrogen), 1x GlutaMax, 1x non-essential amino acids (NEAA, Invitrogen) and 8 ng/mL recombinant human basal fibroblast growth factor (bFGF, Peprotech).

### Alkaline phosphatase (ALP) activity assay

hESCs cultured on 380 Pa, 3.5 kPa, 40 kPa substrates and coverslips for 5 days were subjected to alkaline phosphatase activity analysis with removal of supernatant and addition of an alkaline phosphatase substrate mixture composed of 10 mM diethanolamine (Aladdin), 0.5 mM MgCl_2_ (Aladdin) and 1 mg/ml p-Nitrophenyl phosphate (Sigma-Aldrich) in pH = 10.5 PBS. Cells were incubated in the substrate mixture for 2 h at room temperature. Absorbance was read immediately at 405 nm on a microplate reader (SpectraMax M5, USA, Molecular Devices). ALP activity was normalized to 1 × 10^4^ cells per sample.

### Atomic force microscopy (AFM) for Young’s modulus testing and data analysis

AFM-based mechanical measurements of different cell types were conducted according to our previously published procedures (Wang et al., 2016). Briefly, AFM was performed using the AFM module of Cellhesion200 (JPK instrument, Germany) that is mounted on an inverted optical microscope (Zeiss Observer A1 stand). The AFM probes consisted of a Tipless silicon sensor (ARROW-TL1–50, NANOWORLD) with a modified AFM cantilever which had a nominal spring constant of 0.03 N/m. The cantilever tip was attached with a plain microsphere (6 μm in diameter) to indent the cells for the purpose of simplifying the contact geometry and minimizing the lateral strain of the sample during indentation.

Cells were seeded onto substrates with different stiffness (380 Pa, 3.5 kPa and 40 kPa hydrogels, and coverslip) that were constructed on sterilized 25 mm-diameter coverslips. Prior to cell measurements, the cantilever was first calibrated on the glass coverslip using the thermal vibration method, where the resulting thermal spectrum was fitted with Lorentzian function to determine the spring constant. Individual cells were indented approximately at the center of the cell body (typically sampled over the nucleus) under a piezo-actuated displacement rate of 1 μm/s. All AFM measurements were performed in cell medium at 37 °C.

The Young’s modulus of each cell was obtained by analyzing the force versus indentation curves using JPKSPM Data Processing software with the classical Hertz model, which was valid for small indentations (approximately up to 5–10% of cell height or 200–500 nm). The minimum number of samples measured was 30 individual cells to ensure accurate measurement for each experimental condition (with multiple indentation locations at the center of each cell body). Poisson’s ratio is 0.5 due to the assumption of homogeneity and quasi-incompressibility of cells as previously reported (Caille et al., 2002; Ohayon & Tracqui, 2005).

### Differentiation of hESCs into definitive endoderm (DE) and hepatoblasts (HBs)

hESCs were expanded and also differentiated into DE and HBs for use in subsequent experiments. The hESCs were expanded for 5 days in MEF-conditioned medium (MEF-CM) supplemented with 4 ng/mL bFGF. For seeding onto fabricated hydrogels, hESC colonies were first disassociated into single cells using Accutase (Gibco) suspended in MEF-CM supplemented with 4 ng/mL bFGF (Peprotech) and 10 μM Y27632 (Medchem Express), followed by seeding onto the hydrogels (pre-coated with Matrigel at 4 °C overnight) at a density of 2 × 10^4^/well in 48-well plates.

For the differentiation of hESCs into DE, hESCs that were pre-seeded onto Matrigel-coated plates were treated for 3 days with DE differentiation medium, composed of RPMI 1640 basal medium (Wisent) containing 1x B-27 supplement (Gibco), 100 ng/mL activin A (Peprotech), 3 μM CHIR99021 (Medchem Express) and 1x GlutaMax. For further differentiation into HBs, DE were treated for another 4 days with HB differentiation medium, composed of RPMI 1640 basal medium (Wisent) supplemented with 2% KSR (Invitrogen), 10 ng/mL BMP4 (Peprotech), 10 ng/mL bFGF and 2 mM GlutaMax. Both DE and HB differentiation media were changed daily.

### Re-seeding and expansion of hESC-derived DE and HBs

hESC-derived DE and HBs were treated with Accutase and re-seeded onto hydrogel substrates with varying stiffness, which were coated by 1:50 vitronectin for DE and 1:100 Matrigel for HBs. Cells were re-seeded at a density of 5 × 10^4^/cm^2^ in medium supplemented with 10uM Y27632. Expansion medium for DE contained DMEM/F12 (Gibco) supplemented with 2% KSR, 50 ng/mL bone morphogenetic protein (BMP)-4 (Peprotech), 10 ng/mL epidermal growth factor (EGF; Peprotech), 10 ng/mL vascular endothelial growth factor (VEGF; Peprotech), 50 μg/mL L-ascorbic acid (Sigma-Aldrich), 50 μg/mL Vitamin A (TargetMol), 10 mM nicotinamide (Sigma-Aldrich), 1x GlutaMax and 1% penicillin-streptomycin. Expansion medium for HBs contained DMEM/F12 supplemented with 10% FBS, 1x insulin/transferrin/selenium (ITS, Sigma-Aldrich), 10 mM nicotinamide (Sigma-Aldrich), 10^− 7^ M dexamethasone, 1x GlutaMax, 1% penicillin/streptomycin, 40 ng/mL hepatocyte growth factor (HGF; Peprotech) and 20 ng/mL EGF. Both DE and HB expansion media were changed daily.

### Establishment of hESC-SOX17-GFP and hESC-AFP-mCherry reporter lines

Stable lines of hESC-SOX17-GFP and hESC-AFP-mCherry were obtained through lentivirus-mediated infection and screening. Prior to infection, single cells of hESCs were seeded onto Matrigel-coated well plates in MEF-CM and grown to 40% confluence, following which concentrated SOX17-GFP or AFP-mCherry lentivirus was added to the medium at a final concentration of 8 μg/mL polybrene supplement. After 8 h of infection, hESCs were washed twice with Knockout DMEM and kept in normal MEF-CM.

A screening strategy was used to obtain monoclonal stable cell lines. For this, 2 μg/mL blasticidin (BSD, Sigma-Aldrich) was added for 5 days at 48 h after hESC infection for positive reporter cell screening. Surviving hESCs were then seeded back into the MEF feeder system as single cells until they grew into 10-cell monoclonal colonies. Each monoclonal colony was amplified within a 24-well plate using the MEF feeder system and were differentiated into the DE or HB stage. Colonies that showed GFP and mCherry fluorescence were respectively selected as the monoclonal stable hESC-SOX17-GFP and hESC-AFP-mCherry cell lines.

### Immunostaining, flow cytometry, and real-time quantitative RT-PCR

Immunostaining was performed according to our previously published procedures (Yao et al., 2014). Briefly, cells were fixed in 4% paraformaldehyde for 15 min at room temperature and washed 3 times with PBS. Cells were then permeabilized using 0.5% Triton, and blocked by PBS containing 1% bovine serum albumin (BSA). The cells were incubated with primary antibody (diluted according to the manufacturer’s instructions; see Table 1 for details) at 4 °C overnight, followed by 1 h incubation with the appropriate Dylight secondary antibody (EarthOx Life Sciences, USA). Cell nuclei were marked with Hoechst 33324 (Beyotime). The stained samples were observed and imaged using a Nikon confocal microscope. For F-actin staining, the fixed and permeabilized samples were exposed to 100 nM rhodamine conjugated phalloidin (Cytoskeleton, USA) for 30 min and Hoechst 33342 (Sigma-Aldrich) for 10 min at room temperature, followed by imaging using a Nikon Eclipse Ti-S microscope. ImageJ plot profile tool and Imaris surface tool were used for image quantification.

**Table 1:** Primary antibodies used for flow cytometry and immunofluorescence staining

Primary antibody	Manufacturer	Catalog number
Human/Mouse Oct-3/4 Antibody	R&D	AF1759
Purified anti-human TRA-1-60-R	BioLegend	330,601
Human FOXA2 Antibody	R&D	AF2400
Human SOX17 Antibody	R&D	AF1924
Human/Mouse alpha-Fetoprotein/AFP Antibody	R&D	MAB1368
Anti-PROX1 antibody	Abcam	ab101851
Anti-E Cadherin antibody	Abcam	ab76055
Anti-YAP1 antibody	Abcam	ab52771
Anti-Vinculin antibody	Abcam	ab129002

For flow cytometry, cells were dissociated into single cells using 0.25% Trypsin/EDTA and incubated in blocking buffer containing 1% goat serum for 30 min, followed by incubation in primary and secondary antibodies according to the manufacturers’ instructions. The cells were analyzed using a BD LSRFortessa SORP flow cytometer (BD Biosciences). Three independent samples were examined for each condition, and approximately 10^5^–10^6^ cells were counted for each sample.

For gene expression analysis, total RNA was extracted using TRIzol reagent (Invitrogen), followed by reverse transcription using first-strand cDNA synthesis kit (Takara), according to the manufacturers’ instructions. Expression levels of all genes were quantified using SYBR Green II (Vazyme) on a Bio-Rad CFX96 Real-Time PCR platform, and normalized to the housekeeping gene glyceraldehyde 3-phophate dehydrogenase (*GAPDH*; see Table 2 for primer sequences) using the comparative Ct (2^−ΔΔCT^) method.

**Table 2:** Primer sequences used for real-time RT-PCR

Gene	Forward (5′-3′)	Reverse (5′-3′)
*OCT4*	CTTGAATCCCGAATGGAAAGGG	GTGTATATCCCAGGGTGATCCTC
*NANOG*	TGGGATTTACAGGCGTGAGCCAC	AAGCAAAGCCTCCCAATCCCAAAC
*FOXA2*	GGAGCAGCTACTATGCAGAGC	CGTGTTCATGCCGTTCATCC
*SOX17*	CTCCGGTGTGAATCTCCCC	CACGTCAGGATAGTTGCAGTAAT
*AFP*	TGTACTGCAGAGATAAGTTTAGCTGAC	TCCTTGTAAGTGGCTTCTTGAAC
*PROX1*	CAGATGGAGAAGTACGCAC	CTACTCATGAAGCAGCTCTTG
*GAPDH*	TGCACCACCAACTGCTTAGC	GGCATGGACTGTGGTCATGAG

### Statistical analysis

Statistical analysis was performed in GraphPad Prism using two-way analysis of variance in conjugation with one-way ANOVA and pairwise multiple comparison tests. All data were presented as mean ± standard deviation (SD). Differences were considered statistically significant for *P* < 0.05. Data were obtained from at least three independent samples unless otherwise stated.

## Supplementary information

**Additional file 1: Supplementary Figure 1.** (A) UV exposure time and the corresponding hydrogel Young’s modulus measured by AFM. (B) Surface coated protein characterization by micro-BCA assay for hydrogels of different stiffness. NC represented the coverslip without protein functionalization. ****P < 0.0001. **Supplementary Figure 2.** Numerical values of AYM for epithelial cell types on substrates with different stiffness. AYM of (A, B) MDCK, (C, D) HepaRG, (E, F) hESCs when cultured on substrates with different stiffness at 1, 3 and 5 days, as well as the AYM of parental and daughter cells at day 1. **Supplementary Figure 3.** Numerical values of AYM for mesenchymal cell types on substrates with different stiffness. AYM of (A, B) 3 T3, (C, D) LX-2, (E, F) MSCs when cultured on substrates with different stiffness at 1, 3 and 5 days, as well as the AYM of parental and daughter cells at day 1. **Supplementary Figure 4.** Focal adhesion, cytoskeletal organization and YAP localization in (A) hESC and (B) HepaRG parental cells. **Supplementary Figure 5.** hESCs treated with Y27632 and Blebbistatin showed dissipation of F-actin, suggesting disabled cytoskeletal responses to increase in substrate stiffness. **Supplementary Figure 6.** Significant increase in E-cadherin expression of hESCs during 5 days of in vitro proliferation on a rigid substrate (coverslip).

## Data Availability

The datasets analysed during the current study are available from the corresponding author on reasonable request.

## Abbreviations

AYM: Apparent Young’s modulus
YAP: Yes-associated protein
hESCs: Human embryonic stem cells
DE: Definitive endoderm
HBs: Hepatoblasts
AFM: Atomic force microscopy
