# Correction: Consistent apparent Young’s modulus of human embryonic stem cells and derived cell types stabilized by substrate stiffness regulation promotes lineage specificity maintenance

**DOI:** 10.1186/s13619-025-00252-y

**Published:** 2025-08-18

**Authors:** Anqi Guo, Bingjie Wang, Cheng Lyu, Wenjing Li, Yaozu Wu, Lu Zhu, Ran Bi, Chenyu Huang, Jiao Jiao Li, Yanan Du

**Affiliations:** 1 Department of Biomedical Engineering, Tsinghua-Peking Center for Life Sciences, MOE Key Laboratory of Bioorganic Phosphorus Chemistry and Chemical Biology, School of Medicine, Tsinghua University, Beijing, 100084 China; 2 School of Life Sciences, Tsinghua University, Beijing, 100084 China; 3 Department of Biomedical Engineering, McKelvey School of Engineering, Washington University in St. Louis, St. Louis, 63130 USA; 4 Institute of Systems Engineering, Academy of Military Sciences, Beijing, 100071 China; 5 Department of Dermatology, Beijing Tsinghua Changgung Hospital, School of Clinical Medicine, Tsinghua University, Beijing, 102218 China; 6 Kolling Institute, University of Sydney, Sydney, NSW 2006 Australia

**Correction** **:** **Cell Regeneration 9, 15 (2020)**

**https://doi.org/10.1186/s13619-020-00054-4**

Following publication of the original article (Guo et al. 2020), the authors found two inadvertent errors in Fig. 5b and Fig. S2D. In Fig. 5b, Fig. 6a was mistakenly duplicated during the figure assembly process, and in Fig. S2D, MDCK parental cell AYM data (from Fig. S2B) were incorrectly used as HepaRG parental cell AYM data during plotting. The authors have revisited the original data and have prepared revised versions of Fig. 5 and Fig. S2. It should be noted that in Fig. S2D, due to differences between the current GraphPad software version (GraphPad Prism 10) and the previously used version, the horizontal positions of some scatter points within the 380 Pa, 3.5 kPa, 40 kPa, and coverslip substrate stiffness groups exhibit slight positional variations, while the vertical axis distribution (cell AYM) remains consistent with that of the previous version. The errors were entirely unintentional and do not affect the corresponding conclusion of the study, the authors sincerely apologize and hope a smooth corrigendum to ensure its rigor.

The incorrect Fig. 5 is:

**Fig. 5:**
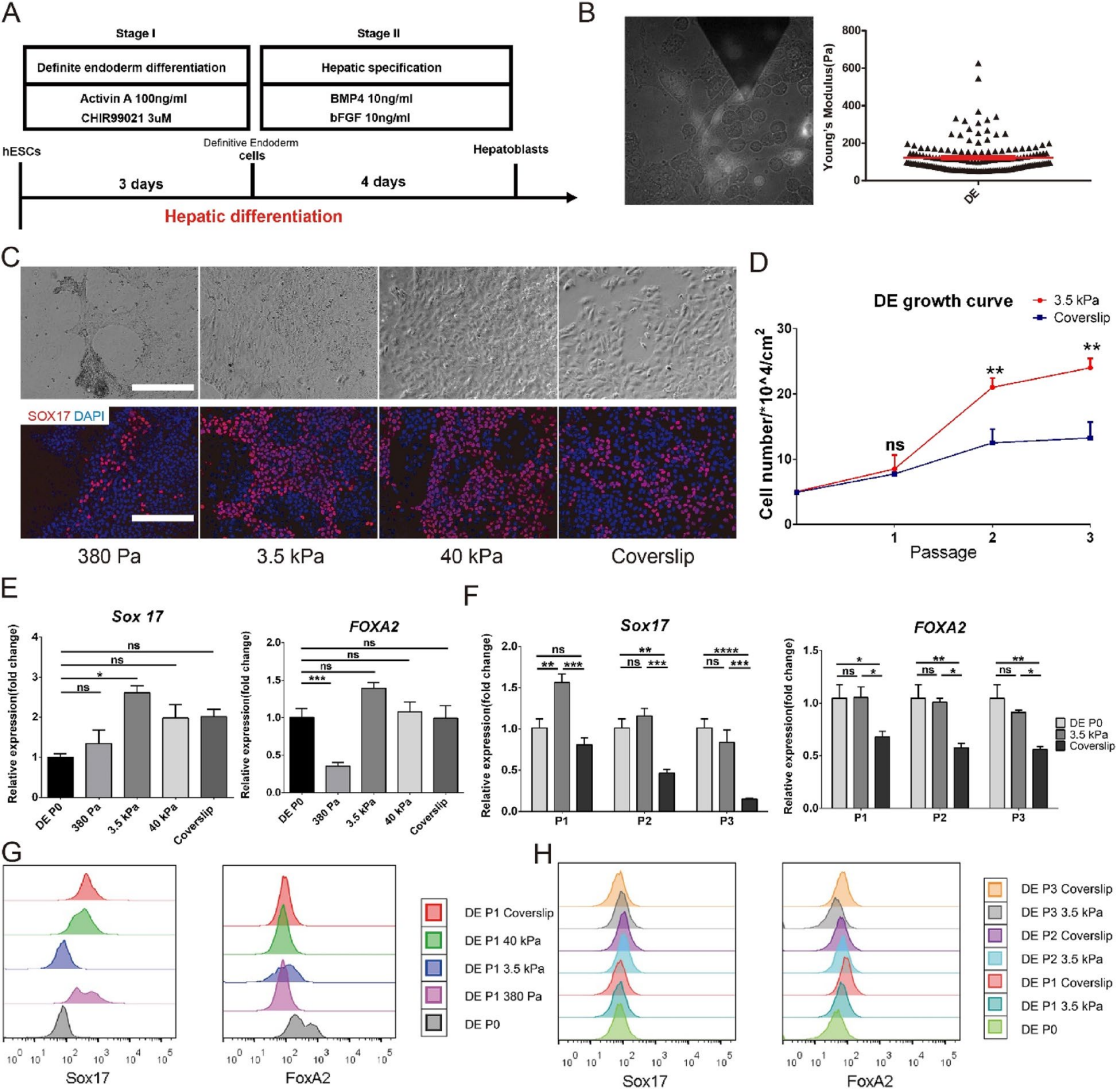
Maintenance of lineage specificity in DE on different substrate stiffness. **a** Schematic of the differentiation strategy for obtaining hESC-derived DE and hepatoblasts. **b** AYM measurement of parental DE with the SOX17-GFP reporter using AFM. **c** Brightfield and immunostaining images of SOX-17 for DE cultured for 5 days on substrates with different stiffness. Scale bars = 200 μm. **d** Growth curve of DE over 3 passages on the 3.5 kPa substrate and coverslip. SOX17 and FOXA2 expression **e** in DE cultured for 5 days on substrates with different stiffness and f in 3 passages of DE on the 3.5 kPa substrate and coverslip. FACS analysis of SOX17 and FOXA2 **g** in DE cultured for 5 days on substrates with different stiffness, and (**h**) in 3 passages of DE on the 3.5 kPa substrate and coverslip. *P < 0.05, **P < 0.01, ***P < 0.001, ****P < 0.0001

The correct Fig. 5 is:

**Fig. 5:**
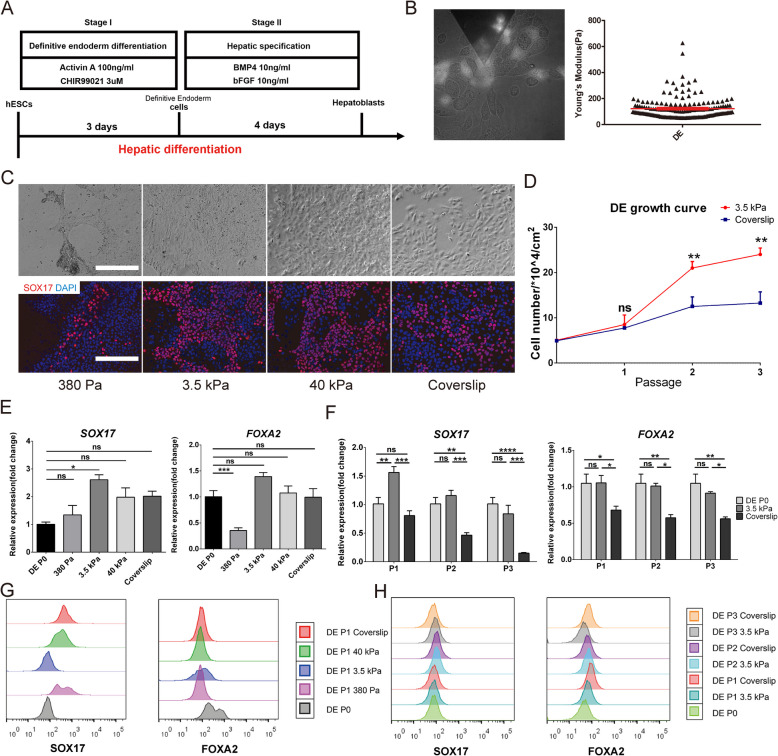
Maintenance of lineage specificity in DE on different substrate stiffness. **a** Schematic of the differentiation strategy for obtaining hESC-derived DE and hepatoblasts. **b** AYM measurement of parental DE with the SOX17-GFP reporter using AFM. **c** Brightfield and immunostaining images of SOX17 for DE cultured for 5 days on substrates with different stiffness. Scale bars = 200 μm. **d** Growth curve of DE over 3 passages on the 3.5 kPa substrate and coverslip. SOX17 and FOXA2 expression **e** in DE cultured for 5 days on substrates with different stiffness and f in 3 passages of DE on the 3.5 kPa substrate and coverslip. FACS analysis of SOX17 and FOXA2 **g** in DE cultured for 5 days on substrates with different stiffness, and (**h**) in 3 passages of DE on the 3.5 kPa substrate and coverslip. *P < 0.05, **P < 0.01, ***P < 0.001, ****P < 0.0001

The original article (Guo et al. 2020) has been corrected.
