# Supplementary material for: Consistent apparent Young’s modulus of human embryonic stem cells and derived cell types stabilized by substrate stiffness regulation promotes lineage specificity maintenance
Source: Cell Regen. 2020 Sep 3;9:15. doi: 10.1186/s13619-020-00054-4 (PMC7467757; doi:10.1186/s13619-020-00054-4)
Supplement: Supplementary file 1 — Additional file 1: Supplementary Figure 1. (A) UV exposure time and the corresponding hydrogel Young’s modulus measured by AFM. (B) Surface coated protein characterization by micro-BCA assay for hydrogels of different stiffness. NC represented the coverslip without protein functionalization. ****P < 0.0001. Supplementary Figure 2. Numerical values of AYM for epithelial cell types on substrates with different stiffness. AYM of (A, B) MDCK, (C, D) HepaRG, (E, F) hESCs when cultured on substrates with different stiffness at 1, 3 and 5 days, as well as the AYM of parental and daughter cells at day 1. Supplementary Figure 3. Numerical values of AYM for mesenchymal cell types on substrates with different stiffness. AYM of (A, B) 3 T3, (C, D) LX-2, (E, F) MSCs when cultured on substrates with different stiffness at 1, 3 and 5 days, as well as the AYM of parental and daughter cells at day 1. Supplementary Figure 4. Focal adhesion, cytoskeletal organization and YAP localization in (A) hESC and (B) HepaRG parental cells. Supplementary Figure 5. hESCs treated with Y27632 and Blebbistatin showed dissipation of F-actin, suggesting disabled cytoskeletal responses to increase in substrate stiffness. Supplementary Figure 6. Significant increase in E-cadherin expression of hESCs during 5 days of in vitro proliferation on a rigid substrate (coverslip). [file 13619_2020_54_MOESM1_ESM.docx]

### Supporting information

1. **Supplementary figures**





**Supplementary Figure 1.** (A) UV exposure time and the corresponding hydrogel Young’s modulus measured by AFM. (B) Surface coated protein characterization by micro-BCA assay for hydrogels of different stiffness. NC represented the coverslip without protein functionalization. ****P<0.0001.


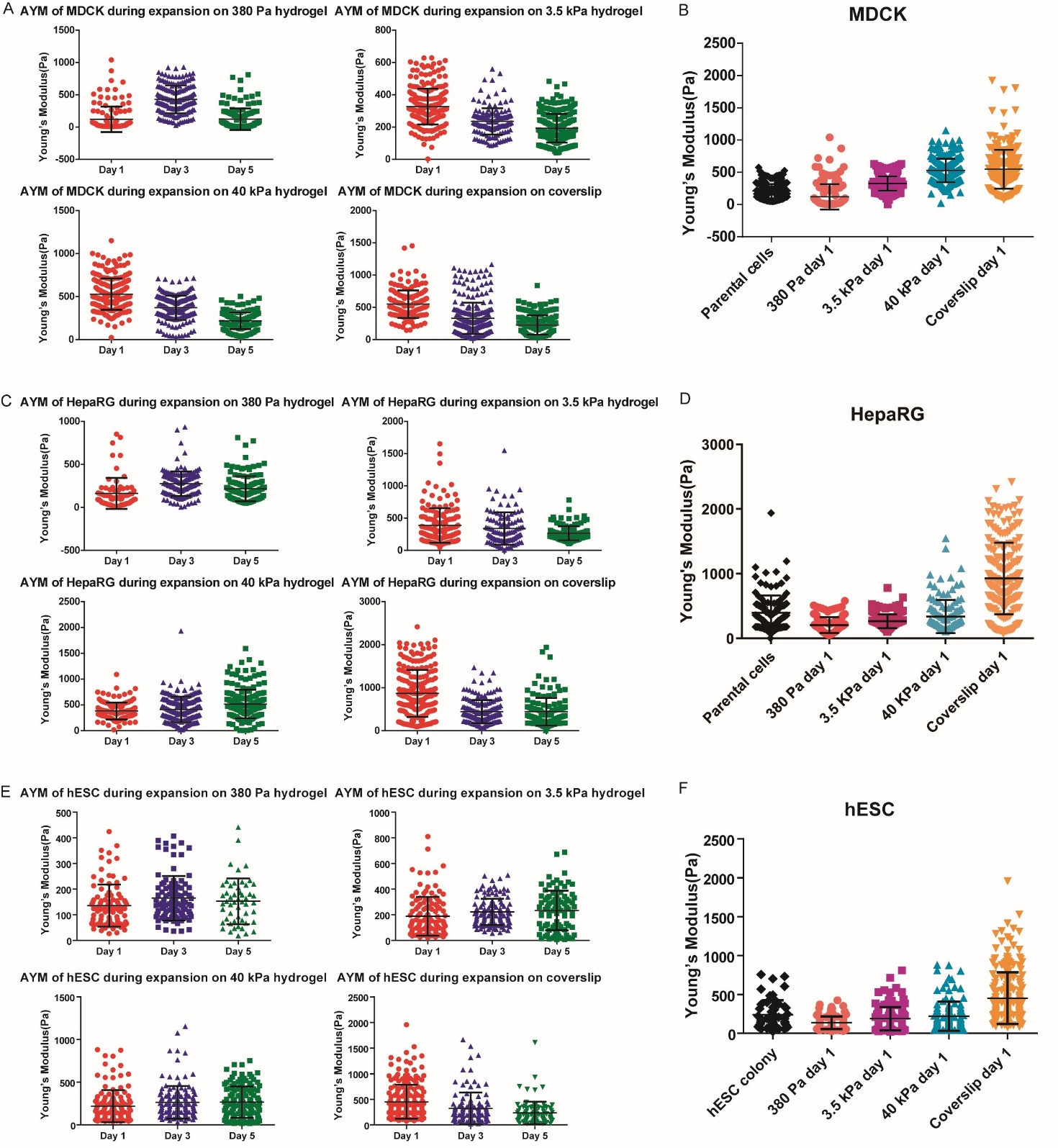


**Supplementary Figure 2.** Numerical values of AYM for epithelial cell types on substrates with different stiffness. AYM of (A, B) MDCK, (C, D) HepaRG, (E, F) hESCs when cultured on substrates with different stiffness at 1, 3 and 5 days, as well as the AYM of parental and daughter cells at day 1.


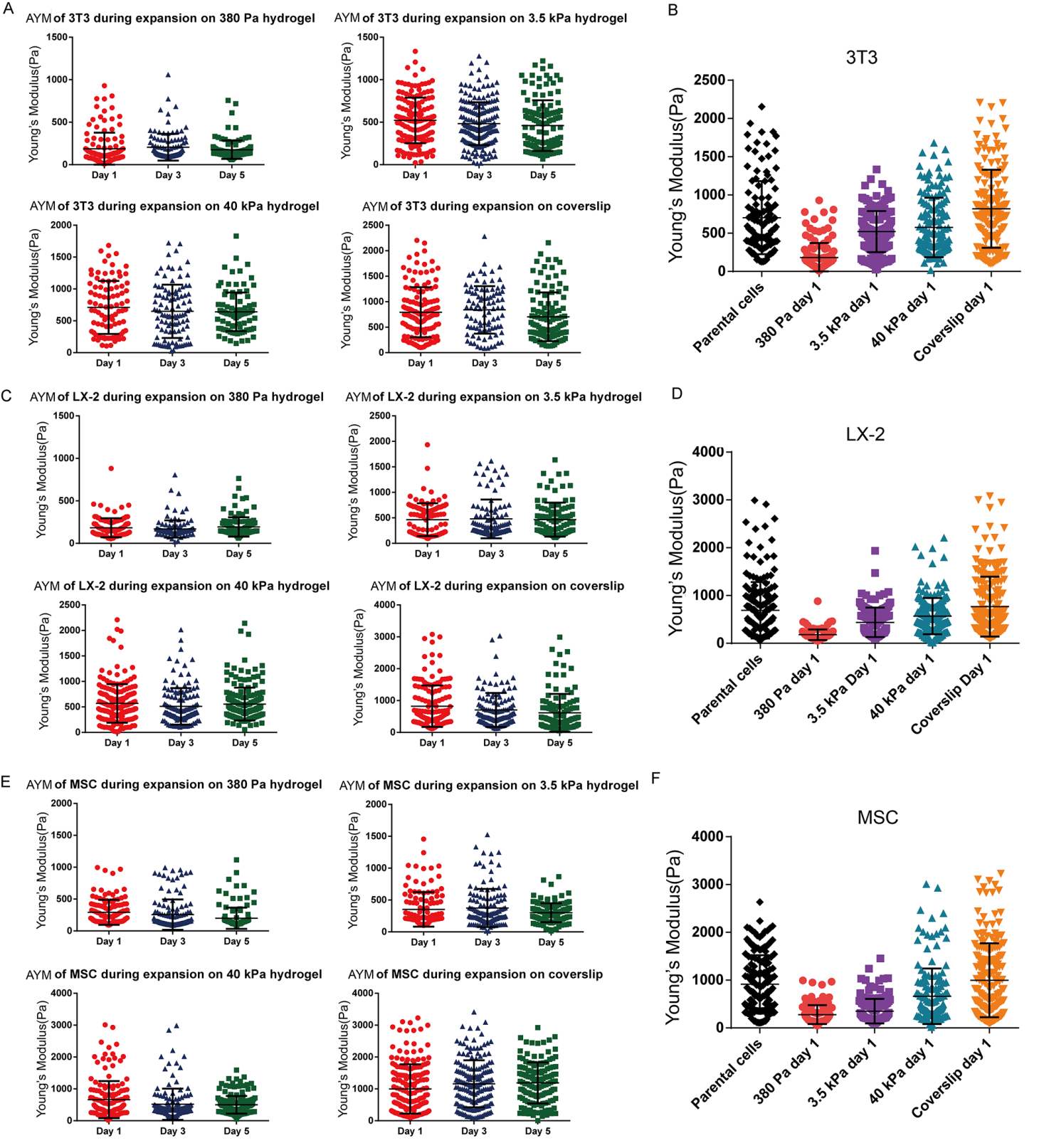


**Supplementary Figure 3.** Numerical values of AYM for mesenchymal cell types on substrates with different stiffness. AYM of (A, B) 3T3, (C, D) LX-2, (E, F) MSCs when cultured on substrates with different stiffness at 1, 3 and 5 days, as well as the AYM of parental and daughter cells at day 1.


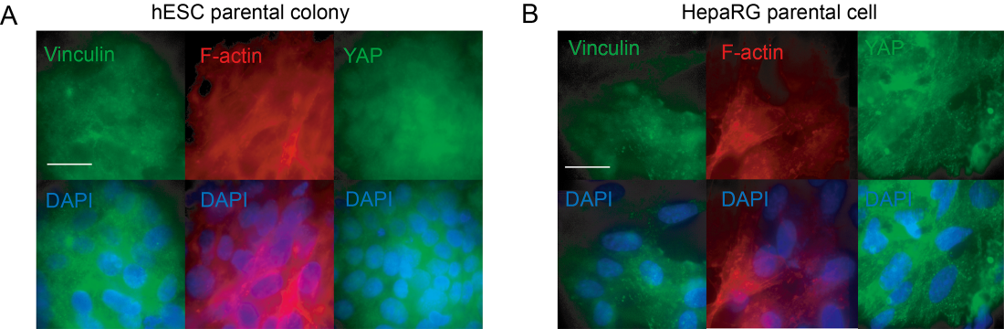


**Supplementary Figure 4.** Focal adhesion, cytoskeletal organization and YAP localization in (A) hESC and (B) HepaRG parental cells.


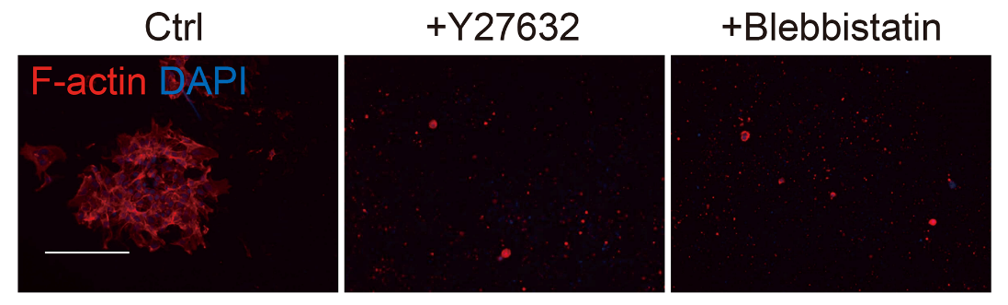


**Supplementary Figure 5.** hESCs treated with Y27632 and Blebbistatin showed dissipation of F-actin, suggesting disabled cytoskeletal responses to increase in substrate stiffness.


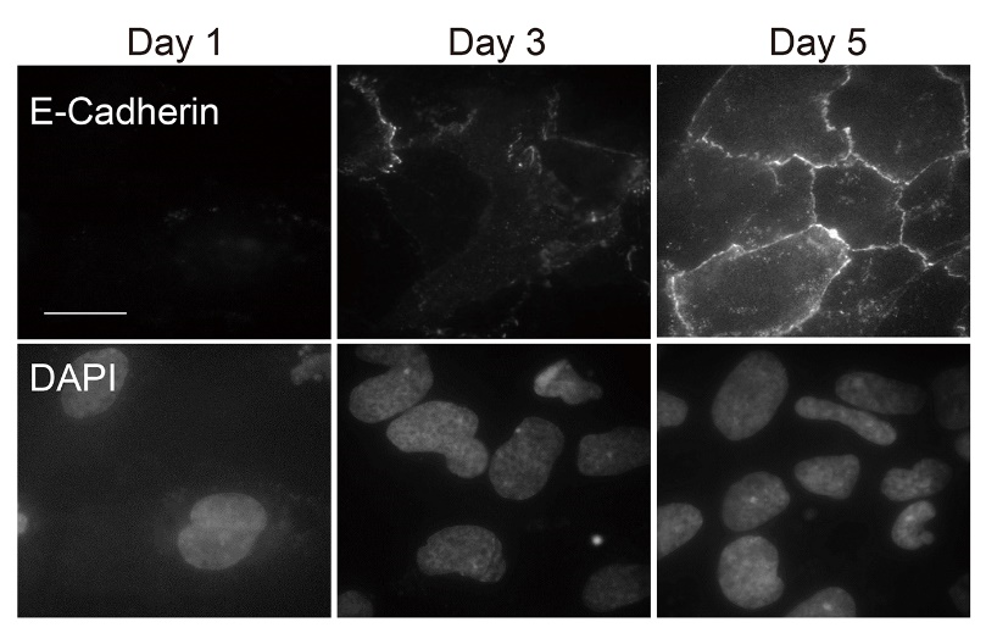


**Supplementary Figure 6.** Significant increase in E-cadherin expression of hESCs during 5 days of *in vitro* proliferation on a rigid substrate (coverslip).
